# Is chest X‐ray still relevant for acute chest syndrome diagnosis?

**DOI:** 10.1002/hsr2.2053

**Published:** 2024-05-01

**Authors:** Yanis Pelinski, Aldiouma Guindo, Christian Kassasseya, Dapa Diallo, Jean‐Benoît Arlet, Armand Mekontso Dessap, Anoosha Habibi, Pablo Bartolucci

**Affiliations:** ^1^ Sickle Cell Referral Center, Department of Internal Medicine Henri‐Mondor University Hospital‐ UPEC, AP‐HP Créteil France; ^2^ Centre de Recherche et de Lutte contre la Drépanocytose Bamako Mali; ^3^ Emergency Department, Hôpital Henri, Mondor Assistance Publique ‐ Hôpitaux de Paris Créteil France; ^4^ Sickle Cell Referral Center, France, Internal Medicine Department, Georges Pompidou European Hospital Assistance Publique‐Hôpitaux de Paris (AP‐HP) Paris France; ^5^ Department of Intensive Care Henri Mondor University Hospital, Assistance Publique Hôpitaux de Paris, Université Paris‐Est Créteil France

**Keywords:** acute chest syndrome, chest X‐ray, sickle cell disease

To the editor,

Over the past 25 to 30 years, the use of ionizing radiation in medicine has greatly increased and a lack of explicit reference to ethics has been recognized. While the benefits of radiological medical procedures far outweigh the radiation risks when these procedures are appropriately prescribed and performed, a substantial fraction of radiation imaging procedures is unjustified or does not provide a net clinical benefit.^1^ From an ethical standing point, the prescription of any medical exam should be governed by four simple questions: what do we expect from the result, does it provide new information, will it change the therapeutic course of action, and does the expected benefit of the result outweigh any short‐term or long‐term adverse effects caused by the exam? This is particularly true for chronic patients, who are frequently hospitalized, in a bid to maximize safety, minimize patient discomfort, as well as reduce medical costs.

Sickle cell disease (SCD) is the most prevalent genetic disease worldwide. Vaso‐occlusive crisis (VOC) is the principal manifestation and first cause of patient hospitalization in SCD. A severe complication of VOC is secondary acute chest syndrome (ACS) which is a major cause of morbidity and mortality in SCD patients.^2^ ACS is characterized by respiratory symptoms and/or fever, especially in children, and is traditionally defined as the appearance of an auscultatory abnormality (crackles and/or bronchial breathing) and/or chest pain, and an infiltrate on chest X‐ray. Pulmonary consolidations, predominantly in the base of the lungs, are the most frequent radiological abnormalities in ACS.^3^

Recent reports suggest that chest X‐ray may not be the most appropriate imaging tool to confirm an ACS diagnosis. Compared to computed tomography (CT) scan, which is considered the gold‐standard imaging tool for lung exploration, bedside chest X‐ray showed good sensitivity but weak specificity.^3^ Moreover, pulmonary artery thrombosis, which occurs in 17% of severe ACS, can only be detected using computed tomography with pulmonary angiogram (CTPA).^4^ Razazi et al. showed that bedside lung ultrasound outperforms bedside chest X‐ray for the diagnosis of lung consolidation and pleural effusion, with a higher sensitivity overall, particularly in postero‐inferior regions, and with high reproducibility.^5^ Bedside ultrasound also has the added benefit of eliminating X‐ray exposure and being cost‐effective. Finally, in the case of an obvious clinical diagnosis with a favorable evolution, the balance between X‐ray, usually with little impact on treatment course, and the risks of repeated irradiation in SCD patients, who are frequently hospitalized, must also be considered.^6^

To determine the major diagnostic tools for ACS, we analyzed the patient cohort of the PRESEV 1 study, a monocenter, prospective, observational study whose aim was to develop a predictive score for ACS during VOC in adult patients with SCD.^7^ ACS was defined as the appearance of an auscultatory abnormality and/or chest pain, associated with an infiltrate on chest X‐ray and/or chest CT, excluding atelectasis. Thoracic imaging was therefore systematically performed in the event of any new auscultatory abnormality or chest pain. A total of 244 patients were included, including 41 in the ACS group. Of these 41 ACS with infiltrates visible on chest X‐ray, lung auscultation showed that 36 (87.8%) patients had either crackles and/or bronchial breathing, three other patients (7.3%) had decreased breath sounds and only two patients (4.9%) had a chest pain without auscultation abnormalities. Of the 203 VOC patients who did not develop ACS, only one (0.5%) had an auscultatory abnormality (crackles) without new radiological infiltrate on chest X‐ray.

To reduce radiation exposure and ensure feasibility in hospitals without easy access to imaging, such as in Africa, we proposed to adapt ACS criteria in the multicenter PRESEV 2 study (NCT03032055). ACS criteria were therefore defined by the appearance of a clear positive auscultatory abnormality (crackles or bronchial breathing), or the presence of chest pain and/or decreased breath sounds with a new radiologic infiltrate. Applying the PRESEV 2 criteria retrospectively to the PRESEV 1 cohort, all patients in the ACS group would have been classified in the same way (sensitivity 100%; 41/41) and one patient among the 241 in the VOC group would have been wrongly classified in the ACS group (specificity 99.5%, 240/241) (Table 1).

**Table 1:** PRESEV 2 ACS criteria applied to PRESEV 1 cohort.

	PRESEV 1 VOC	PRESEV 1 ACS	Total PRESEV 1 cohort
Number (%)	203 (83.2)	41 (16.8)	244 (100)
New crackles, *n* (%)	1 (0.5)	33 (80.5)	35 (14.3)
New bronchial breathing, *n* (%)	0	7 (17.1)	7 (2.9)
Both, *n* (%)	0	4 (9.8)	4 (1.6)
**Total criterion A, *n* (%)**	**1** (**0.5)**	**36** (**87.8)**	**38** (**15.6)**
Chest pain, *n* (%)	1 (0.5)	33 (80.5)	34 (13.9)
Decreased breath sounds, *n* (%)	0	13 (31.8)	13 (5.3)
Both clinical abnormalities, *n* (%)	0	12 (29.2)	12 (4.9)
New radiological infiltrate, *n* (%)^a^	0	41 (100)	41 (16.8)
**Total criterion B, *n* (%)**	**0**	**34** (**82.9)**	**34** (**13.9)**
**Both criteria A and B, *n* (%)**	**0**	**29** (**70.7)**	**30** (**12.3)**
**Total ACS with PRESEV 2 criteria, *n* (%)**	**1** (**0.5)**	**41** (**100)**	**42** (**17.2)**

Abbreviations: ACS, acute chest syndrome; CT, computed tomography; VOC, vaso‐occlusive crisis.

Criterion A: appearance of an auscultatory abnormality (crackles or bronchial breathing).

Criterion B: association of a new radiologic infiltrate, and chest pain or decreased breath sounds.

Results are expressed as *n* (%).

^a^ X‐rays were only taken in cases of abnormal auscultation or chest pain.

Our results suggest that pulmonary auscultation is a robust diagnostic tool for ACS. In the presence of a new clearly positive auscultatory anomaly, a chest X‐ray does not provide additional significant information justifying subjecting the patient to radiation exposure. Regardless, chest X‐ray remains commonly used to diagnose ACS. Over the last 2 years, 1860 patients were hospitalized for VOC at Henri Mondor hospital, of whom 720 (38.7%) had a chest X‐ray. We therefore propose a new algorithm for the diagnosis of ACS (Figure 1), primarily based on lung auscultation, the risk for pulmonary artery thrombosis,^8^ and clinical severity as defined by the French guidelines.^6^ The algorithm promotes the use of imaging only when clinically relevant, and favors the use of ultrasound over X‐ray, except for patients at risk for pulmonary artery thrombosis or with clinical severity, that require CTPA. The role of the CTPA in this algorithm probably makes it not universally applicable in its current form, and some indications may be subject to discussion based on local practices. Nevertheless, by significantly reducing the need for imaging, it could contribute to a global standardization of practices. Finally, considering that hospitalizations for VOC are frequent and ACS is a recurrent condition, this algorithm could enhance ACS diagnosis while effectively reducing patient exposure to irradiation throughout their lifetime.

**Figure 1:**
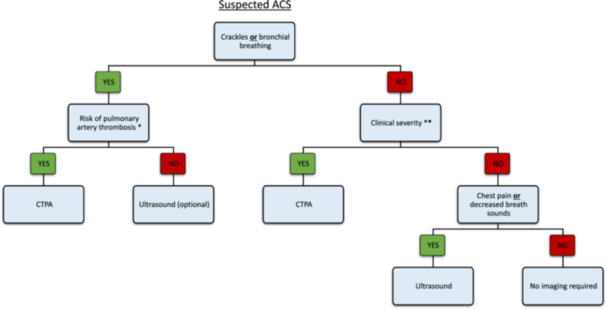
Algorithm for acute chest syndrome (ACS) diagnosis in patients with vaso‐occlusive crisis. *Pulmonary artery thrombosis risk factors: lack of a triggering factor for ACS, baseline hemoglobin >82 g/L, platelet count > 440×10^9^/L and a PaCO_2_ < 38 mmHg at ACS diagnosis. **Clinical severity according to French guidelines: Respiration rate >30/min or <10/min, trouble speaking, altered level of consciousness, extended auscultatory abnormalities, associated cardiac or renal insufficiency, cardiac frequency >120 bpm, arterial oxygen pressure PaO_2_ <60 mmHg in ambient air, arterial carbon dioxide PaCO_2_ <50 mmHg, O2 requirement >4 L/min to obtain SpO_2_ <98%. CTPA, computed tomography with pulmonary angiogram.

## AUTHOR CONTRIBUTIONS

**Yanis Pelinski**: Data curation; writing—original draft. **Aldiouma Guindo**: Investigation; writing—review and editing. **Christian Kassasseya**: Investigation; writing—review and editing. **Dapa Diallo**: Investigation; writing—review and editing. **Jean‐Benoît Arlet**: Investigation; writing—review and editing. **Armand Mekontso Dessap**: Investigation; writing—review and editing. **Anoosha Habibi**: Investigation; writing—review and editing. **Pablo Bartolucci**: Conceptualization; writing—review and editing.

## CONFLICT OF INTEREST STATEMENT

The authors declare no conflicts of interest.

## TRANSPARENCY STATEMENT

The lead author Pablo Bartolucci affirms that this manuscript is an honest, accurate, and transparent account of the study being reported; that no important aspects of the study have been omitted; and that any discrepancies from the study as planned (and, if relevant, registered) have been explained.

## Data Availability

Data sharing is not applicable to this article as no new data were created or analyzed in this study.
